# Supplementary material for: DupyliCate: mining, classifying, and characterizing gene duplications
Source: Sci Rep. 2026 May 28;16:16557. doi: 10.1038/s41598-026-55350-x (PMC13219399; doi:10.1038/s41598-026-55350-x)
Supplement: Supplementary file 2 — Supplementary Material 2 [file 41598_2026_55350_MOESM2_ESM.pdf]

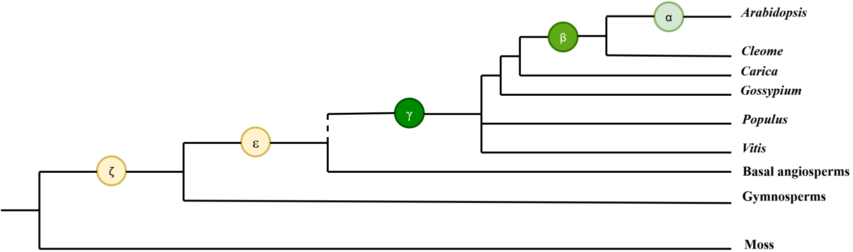

Phylogenetic tree depicting the major polyploidization events (γ,β,α) in the evolutionary history leading to *Arabidopsis* along with the ancient polyploid events ζ and ε reported in the evolutionary trajectory leading to seed plants and angiosperms, respectively. The dotted line before the γ is placed to show the other polyploidization events (τ, σ, ρ) not included in the representation [27, 29].
