# Supplementary material for: DupyliCate: mining, classifying, and characterizing gene duplications
Source: Sci Rep. 2026 May 28;16:16557. doi: 10.1038/s41598-026-55350-x (PMC13219399; doi:10.1038/s41598-026-55350-x)
Supplement: Supplementary file 4 — Supplementary Material 4 [file 41598_2026_55350_MOESM4_ESM.pdf]

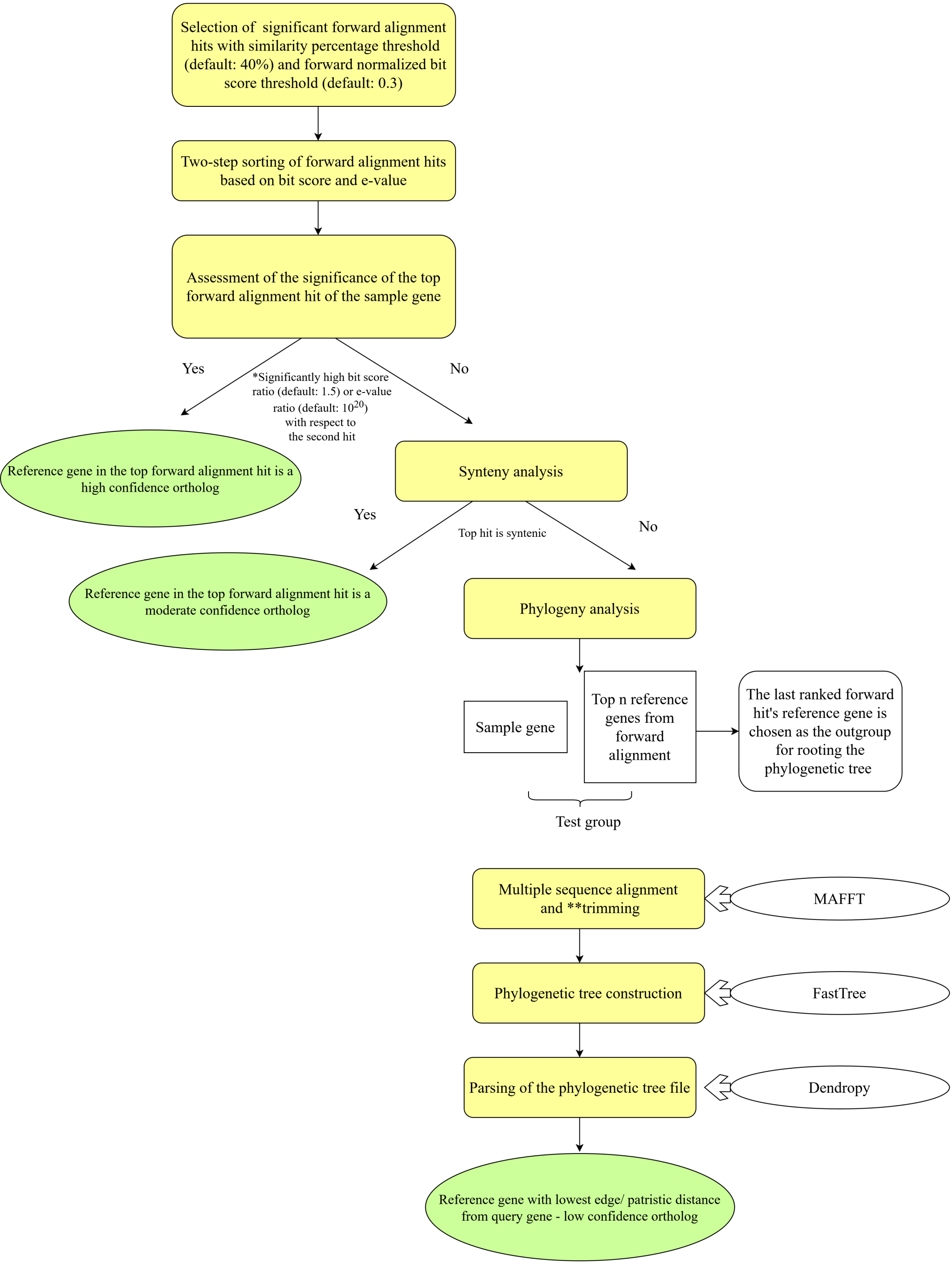

Ortholog assignment steps in the presence of a reference organism. *The bit score and e-value ratios used for checking if the top hit is significantly different from the second hit, were empirically determined. **Trimming of multiple sequence alignment files is performed by code in-built within DupyliCate and does not rely on any external tool for this step.
