# Supplementary material for: DupyliCate: mining, classifying, and characterizing gene duplications
Source: Sci Rep. 2026 May 28;16:16557. doi: 10.1038/s41598-026-55350-x (PMC13219399; doi:10.1038/s41598-026-55350-x)
Supplement: Supplementary file 5 — Supplementary Material 5 [file 41598_2026_55350_MOESM5_ESM.pdf]

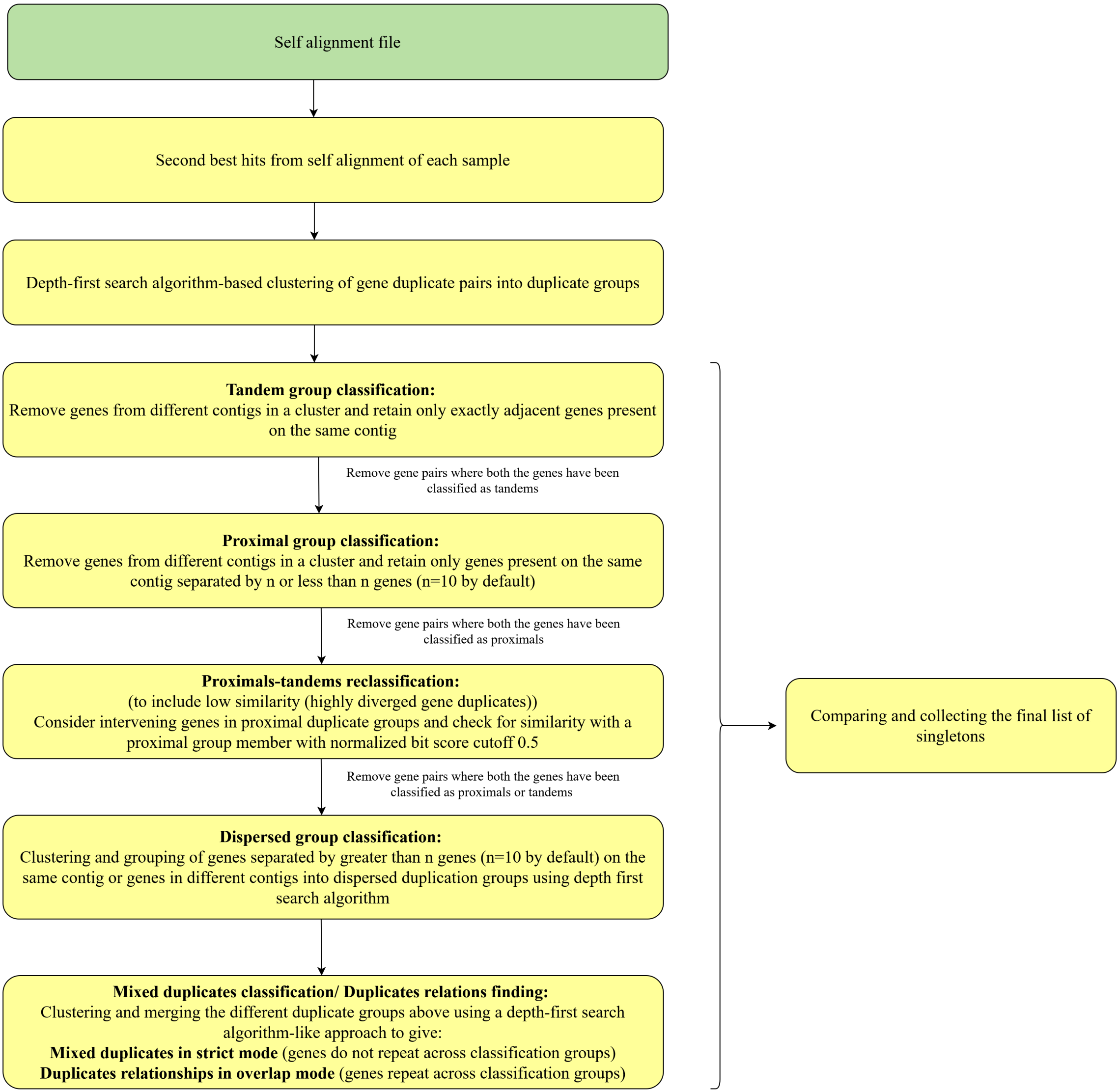

Flow chart of the algorithms and steps involved in gene duplicates array clustering, and classification
