# Supplementary material for: DupyliCate: mining, classifying, and characterizing gene duplications
Source: Sci Rep. 2026 May 28;16:16557. doi: 10.1038/s41598-026-55350-x (PMC13219399; doi:10.1038/s41598-026-55350-x)
Supplement: Supplementary file 7 — Supplementary Material 7 [file 41598_2026_55350_MOESM7_ESM.pdf]

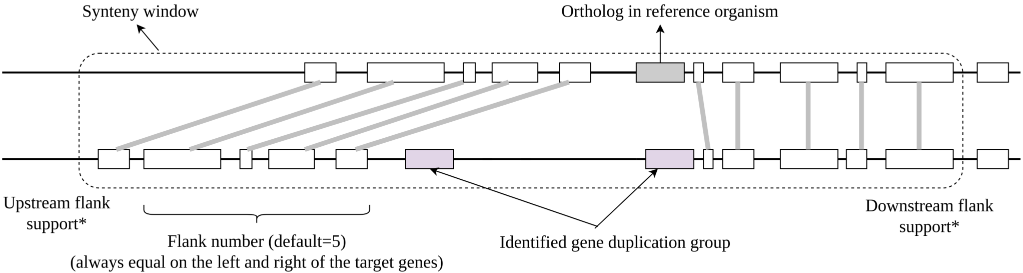

* - sufficient flanking genes must be best hits in forward local alignment
(default flank support is 1 each in the upstream and downstream flank regions)

Schematic representation of synteny analysis performed for small scale gene duplicates as well as ortholog assignment
