# Supplementary material for: DupyliCate: mining, classifying, and characterizing gene duplications
Source: Sci Rep. 2026 May 28;16:16557. doi: 10.1038/s41598-026-55350-x (PMC13219399; doi:10.1038/s41598-026-55350-x)
Supplement: Supplementary file 8 — Supplementary Material 8 [file 41598_2026_55350_MOESM8_ESM.pdf]

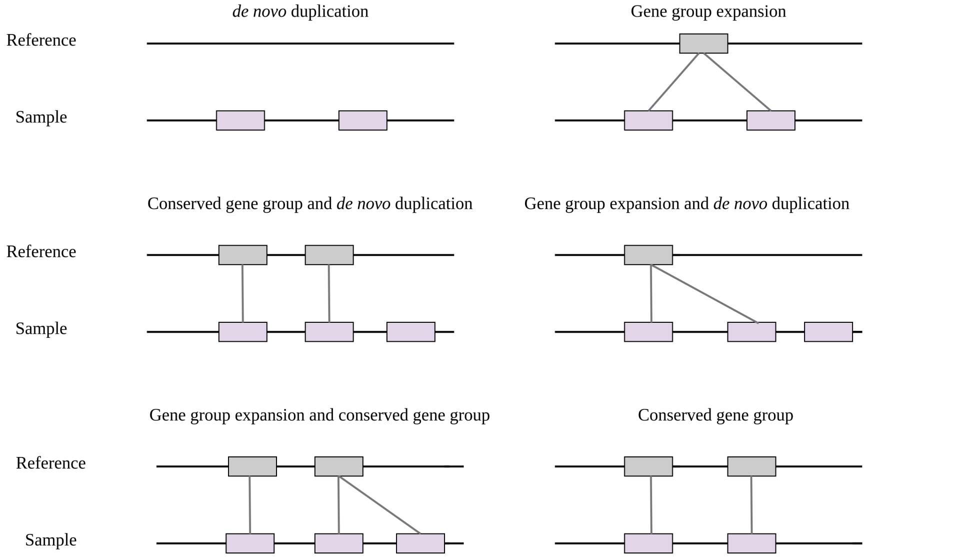

Schematic representation of the different possible types of the identified small scale gene duplicate groups with respect to orthologous genes in the reference organism
