# Supplementary material for: DupyliCate: mining, classifying, and characterizing gene duplications
Source: Sci Rep. 2026 May 28;16:16557. doi: 10.1038/s41598-026-55350-x (PMC13219399; doi:10.1038/s41598-026-55350-x)
Supplement: Supplementary file 9 — Supplementary Material 9 [file 41598_2026_55350_MOESM9_ESM.pdf]

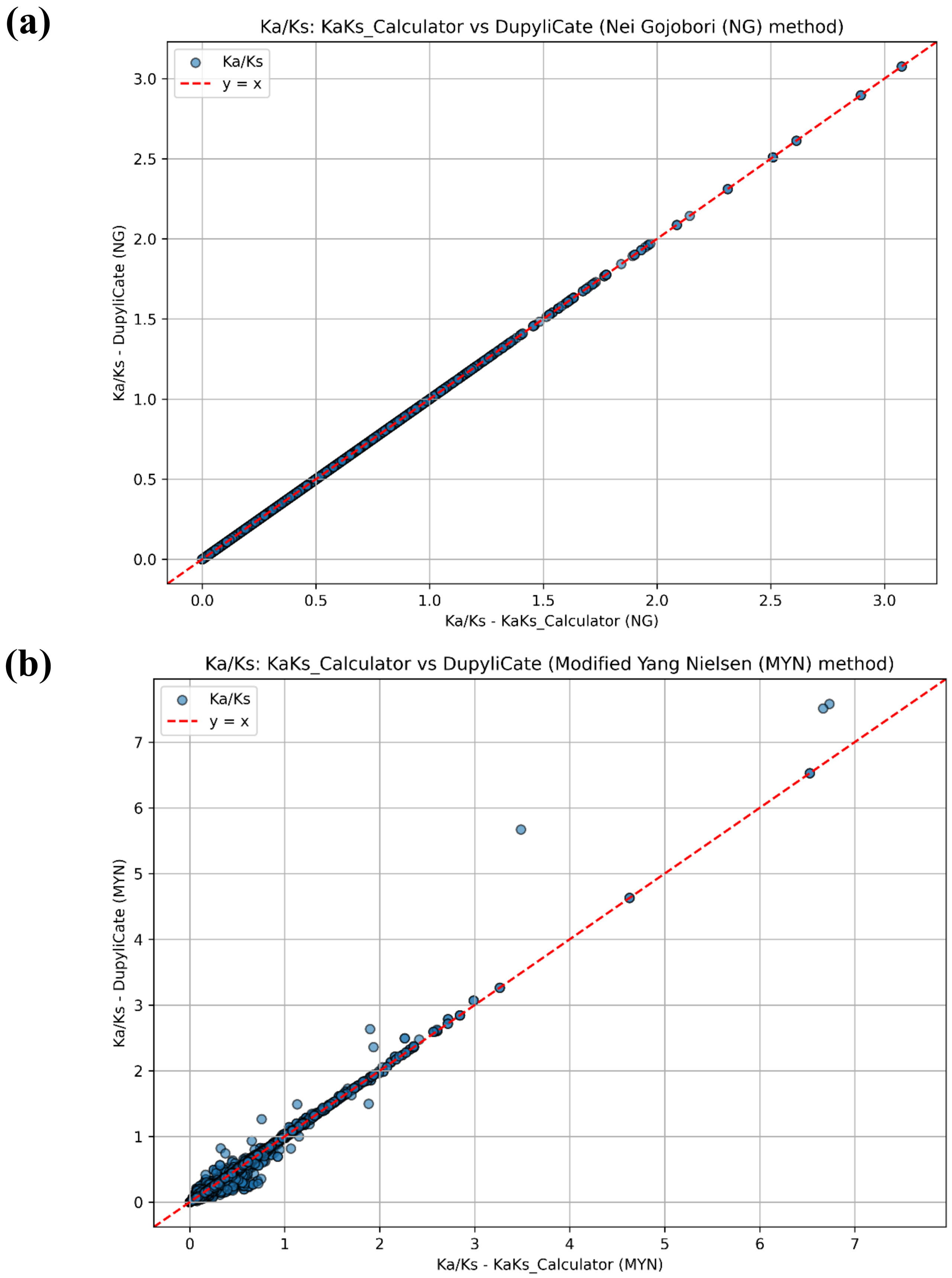

Correlation analysis of KaKs results using python implementations of the (a) Nei Gojobori (NG) method with the results of NG method in KaKs_Calculator2.0 and the (b) Modified Yang Nielsen (MYN) method with the results of MYN method in KaKs_Calculator2.0
