# Supplementary figures and images for: DupyliCate: mining, classifying, and characterizing gene duplications
Source: Sci Rep. 2026 May 28;16:16557. doi: 10.1038/s41598-026-55350-x (PMC13219399; doi:10.1038/s41598-026-55350-x)

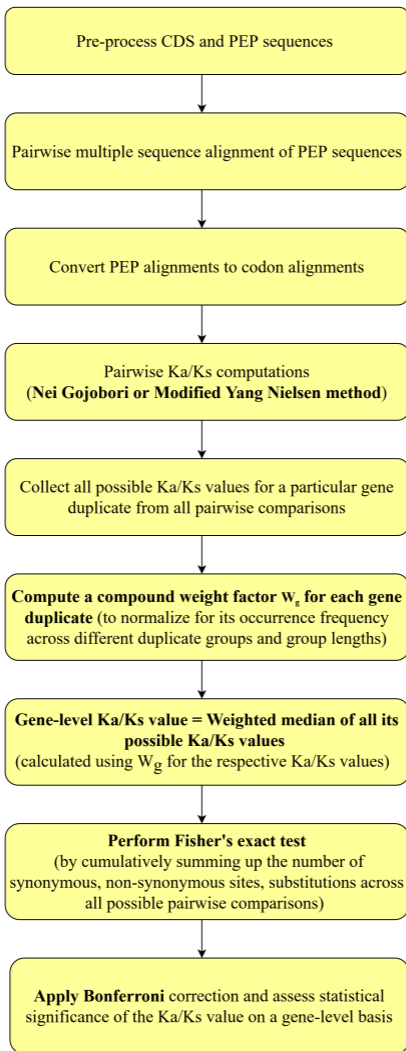

Steps involved in gene-level Ka/Ks computation in DupyliCate

Supplement: Supplementary file 10 — Supplementary Material 10 [file 41598_2026_55350_MOESM10_ESM.pdf]

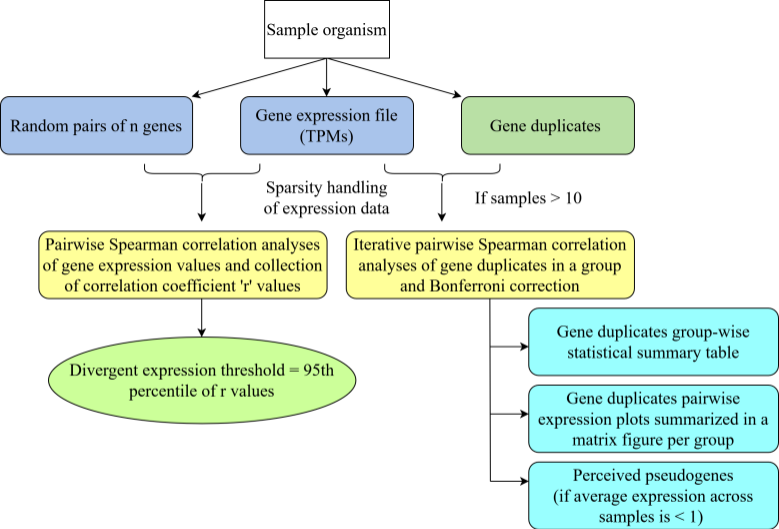

Methodology of statistical analysis and gene expression analysis of gene duplicates

Supplement: Supplementary file 11 — Supplementary Material 11 [file 41598_2026_55350_MOESM11_ESM.pdf]
