# Supplementary material for: DupyliCate: mining, classifying, and characterizing gene duplications
Source: Sci Rep. 2026 May 28;16:16557. doi: 10.1038/s41598-026-55350-x (PMC13219399; doi:10.1038/s41598-026-55350-x)
Supplement: Supplementary file 12 — Supplementary Material 12 [file 41598_2026_55350_MOESM12_ESM.pdf]

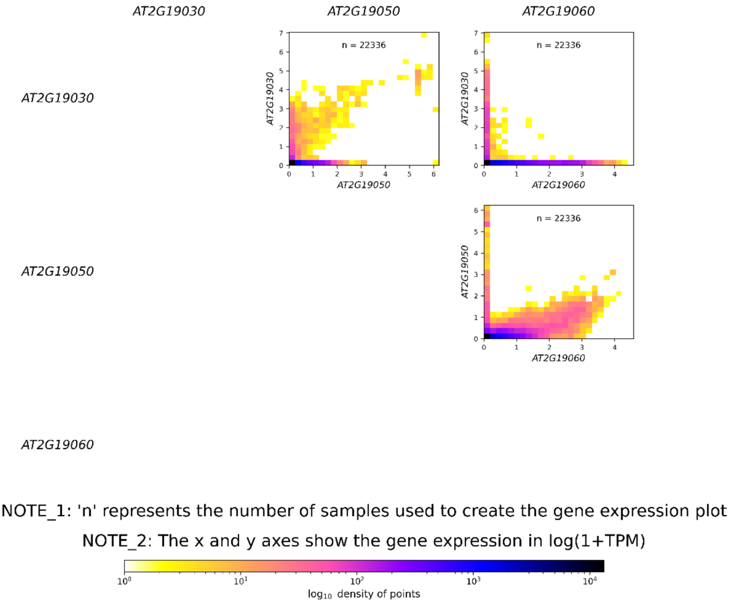

Matrix type gene expression plot
